# Supplementary material for: A Review of Selective Laser Trabeculoplasty: “The Hype Is Real”
Source: J Clin Med. 2022 Jul 4;11(13):3879. doi: 10.3390/jcm11133879 (PMC9267824; doi:10.3390/jcm11133879)
Supplement: Supplementary file 1 [file jcm-11-03879-s001.zip › jcm-1677302-supplementary.pdf]

Supplementary materials

Search strategy in PubMed database, using PubMed search builder and MeSH terms where applicable

(((selective[All Fields] AND ("lasers"[MeSH Terms] OR "lasers"[All Fields] OR "laser"[All Fields]) AND ("trabeculectomy"[MeSH Terms] OR "trabeculectomy"[All Fields] OR "trabeculoplasty"[All Fields])) OR "slt"[All Fields]) OR (("lasers"[MeSH Terms] OR "lasers"[All Fields] OR "laser"[All Fields]) AND ("trabeculectomy"[MeSH Terms] OR "trabeculectomy"[All Fields] OR "trabeculoplasty"[All Fields]))) AND (((((("glaucoma, open-angle"[MeSH Terms] OR ("glaucoma"[All Fields] AND "open- angle"[All Fields]) OR "open-angle glaucoma"[All Fields] OR ("open"[All Fields] AND "angle"[All Fields] AND "glaucoma"[All Fields]) OR "open angle glaucoma"[All Fields]) OR ("glaucoma, open- angle"[MeSH Terms] OR ("glaucoma"[All Fields] AND "open-angle"[All Fields]) OR "open-angle glaucoma"[All Fields] OR ("open"[All Fields] AND "angle"[All Fields] AND "glaucoma"[All Fields]) OR "open angle glaucoma"[All Fields])) OR ("ocular hypertension"[MeSH Terms] OR ("ocular"[All Fields] AND "hypertension"[All Fields]) OR "ocular hypertension"[All Fields])) OR OAG[All Fields]) OR OHT[All Fields]) OR (exfoliative[All Fields] AND ("glaucoma"[MeSH Terms] OR "glaucoma"[All Fields]) OR OR (pigmentary[All Fields] AND ("glaucoma"[MeSH Terms] OR "glaucoma"[All Fields]))) OR XFG[All Fields])

Number of results: 2032; when restricted to the last 5 years: 377.

Number of clinical trials and meta-analyzes and randomized controlled trials: 310; when restricted to 5 years: 49.

When applicable, we cited primary sources, excluding the restriction to 5 years.
